# Quantitative Analysis of Oat (*Avena sativa* L.) and Pea (*Pisum sativum* L.) Saponins in Plant-Based Food Products by Hydrophilic Interaction Liquid Chromatography Coupled with Mass Spectrometry

**DOI:** 10.3390/foods12050991

**Published:** 2023-02-26

**Authors:** Anastassia Bljahhina, Dmitri Pismennõi, Tiina Kriščiunaite, Maria Kuhtinskaja, Eeva-Gerda Kobrin

**Affiliations:** 1 Center of Food and Fermentation Technologies (TFTAK), Mäealuse 2/4, 12618 Tallinn, Estonia; 2 Department of Chemistry and Biotechnology, Tallinn University of Technology, Akadeemia Tee 15, 12618 Tallinn, Estonia

**Keywords:** oat, pea, avenacosides, saponin B, DDMP saponin, plant-based protein

## Abstract

This work presents the sample extraction methods for solid and liquid sample matrices for simultaneous quantification of oat (*Avena sativa* L.) and pea (*Pisum sativum* L.) saponins: avenacoside A, avenacoside B, 26-desglucoavenacoside A, and saponin B and 2,3-dihydro-2,5-dihydroxy-6-methyl-4H-pyran-4-one (DDMP) saponin, respectively. The targeted saponins were identified and quantified using a hydrophilic interaction liquid chromatography with mass spectrometric detection (HILIC-MS) method. The simple and high-throughput extraction procedure was developed for solid oat- and pea-based food samples. In addition, a very simple extraction procedure for liquid samples, without the need to use lyophilisation, was also implemented. Oat seed flour (U-^13^C-labelled) and soyasaponin Ba were used as internal standards for avenacoside A and saponin B, respectively. Other saponins were relatively quantified based on avenacoside A and saponin B standard responses. The developed method was tested and successfully validated using oat and pea flours, protein concentrates and isolates, as well as their mixtures, and plant-based drinks. With this method, the saponins from oat- and pea-based products were separated and quantified simultaneously within 6 min. The use of respective internal standards derived from U-^13^C-labelled oat and soyasaponin Ba ensured high accuracy and precision of the proposed method.

## 1. Introduction

The demand for sustainable protein sources in food production is continuously growing [1,2]. Oat (*Avena sativa* L.) and pea (*Pisum sativum* L.) proteins in the form of concentrates or isolates can act as an alternative to animal proteins due to their potential ability to provide desirable technological properties in plant-based meat and dairy substitutes [3,4]. Pea protein is an insufficient source of methionine but, on the other hand, has a high content of the essential amino acid lysine and branched-chain amino acids-leucine, isoleucine, and valine [4]. In contrast to pea, oat contains enough methionine but a scarce amount of lysine [3]. Blending oat and pea proteins in products is one way to achieve a complete essential amino acid profile [5], and such products are already available on the market. However, one of the main obstacles in the application of plant-based proteins in food production is their bitter and astringent off-taste [6,7,8]. It has been suggested that saponins might be the main cause of this sensation [9,10,11,12,13,14] influencing consumer acceptance.

**Figure 1:**
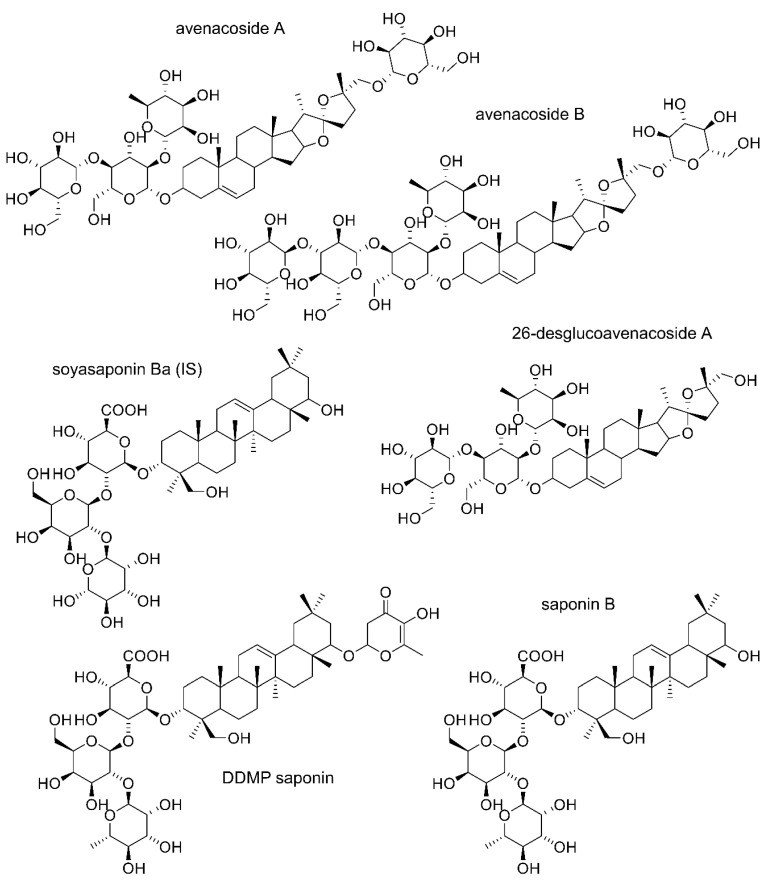
Chemical structures of saponins: avenacoside A, avenacoside B, and 26-desglucoavenacoside A from oat, saponin B and DDMP from pea, and soyasaponin Ba (used as internal standard [IS]).

Saponins are a diverse group of secondary defence metabolites widely spread in plant species [15]. Saponins investigated in this study are amphiphilic molecules, with polar water-soluble sugar moieties attached to a nonpolar, water-insoluble steroid or triterpene core [16]. Oats, as the only cereals capable of accumulating saponins, contain bisdesmosidic steroidal saponins avenacoside A and B, and monodesmosidic 26-desglucoavenacoside A in their leaves and grains (Figure 1) [9,10,12,13,17]. Saponin B and 2,3-dihydro-2,5-dihydroxy-6-methyl-4H-pyran-4-one (DDMP) saponin are monodesmosidic triterpenoid saponins found in peas [18,19]. Besides being taste-active bitter compounds, saponins have also been reported as antinutrients. As such, they may affect nutrient absorption by inhibiting metabolic and digestive enzymes [20] and by binding to minerals such as zinc and iron [21]. High concentrations of saponins in the diet may lead to hypocholesterolemic effect [22], hypoglycemia [23], inefficient protein digestion, vitamin and mineral uptake in the gut, and the development of a leaky gut [24]. Despite the reported negative nutritional impact, some studies have also shown positive cholesterol-lowering [25] and anticancerogenic [26] effects of saponins.

The analysis of saponins could be performed using a wide range of classical methods such as gravimetry [15,27], hemolysis [28], bioassays [29], and spectrophotometry [30]. In addition, different saponins could be separated and analysed using chromatographic methods, e.g., thin-layer chromatography [31,32], gas chromatography [33], and high-performance liquid chromatography (HPLC) [19]. The detection of the saponin class compounds could be carried out using the simplest optical detection methods [34,35], but these methods usually lack the selectivity and sensitivity of more advanced analytical techniques such as mass spectrometry [9,17,36,37,38]. The saponin extraction and the pre- and post-extraction sample clean-up before the LC analysis [9,17,19,39] are required to obtain a clean extract which would minimise matrix effect in mass spectrometric measurements. However, these sample preparation procedures are time-consuming and unsuitable for routine analysis of large amounts of samples. This creates the need for an improved, efficient, sensitive, more selective, and reproducible extraction method of saponins prior to the analysis. The use of liquid chromatography coupled with mass spectrometry (LC-MS) allows more precise and selective determination of the contents of different types of saponins in various plant species: oat [9,13,17], pea [19,32], and soya [39,40]. Although, the amounts of saponins have been quantified mainly from the seeds or husks of numerous oat and pea varieties [9,19,32], there is a lack of data concerning the concentrations of saponins in processed food ingredients, and the half- and end-products produced therefrom. To the best of our knowledge, there is no versatile method for the determination of saponins derived from different plant species in various food matrices.

The objective of this study was to develop simple sample extraction methods for solid and liquid plant-based food sample matrices for the selective and quantitative determination of five oat and pea saponins: avenacoside A, avenacoside B, 26-desglucoavenacoside A, saponin B, and DDMP saponin, using hydrophilic interaction liquid chromatography with mass spectrometric detection (HILIC-MS). To our knowledge, there are no reports on simultaneous HILIC analysis of the above-mentioned saponins in solid and liquid samples containing concurrent oat and pea ingredients.

## 2. Materials and Methods

### 2.1. Chemicals and Materials

HPLC-grade acetonitrile (MeCN), methanol (MeOH), ethanol (EtOH), hexane, propan-2-ol (IPA), and formic acid (FA) (for MS, 98%) were purchased from Honeywell (Charlotte, NC, USA). The standard compounds avenacoside A, saponin B (soyasaponin I), and soyasaponin Ba phyproof^®^ were purchased from Sigma-Aldrich (Darmstadt, Germany). Uniformly isotopically labelled oat seed flour (U-^13^C oat seeds, *Avena sativa* 97 atom%) was obtained from IsoLife BV (Wageningen, The Netherlands). Ultrapure water (18.2 MΩ·cm) was prepared with MilliQ^®^ HX 7040SD equipped with MilliQ LC-Pak (Merck KGaA, Darmstadt, Germany). Biotage Isolute^®^ PLD+ and C18 columns (100 mg/1 mL) were purchased from Biotage Sweden AB (Uppsala, Sweden). Amicon Ultra-0.5 centrifugal filter units (3, 10, 30, 50 kDa) and Millex-LCR filters (pore size 0.2 µm, filter dimension 13 mm) were obtained from Merck KgaA (Darmstadt, Germany).

### 2.2. Food Samples

Yellow pea flour, whole-grain oat flour, and oat and pea drinks were purchased from a local supermarket. Pea protein isolate (Bang & Bonsomer Estonia OÜ, Tallinn, Estonia), pea protein concentrate (Aloja-Starkelsen Ltd., Limbažu novads, Latvia), and oat protein concentrate (Lantmännen, Stockholm, Sweden) were obtained from producers. The composition and nutritional information available on the product label of these products is available in Supplementary Table S1. Untreated and extruded blends of pea protein isolate, oat protein concentrate, and pea protein concentrate (52:28:20, *w*/*w*) were produced in-house by following a previously published protocol [41].

### 2.3. Extraction Method for Solid Samples and for Liquid Samples

Sample extraction methods 1A, 1B, 2A, 2B, and 2C, which were tested during solid sample extraction method development, are described in the supplementary information.

Solid sample extraction (method 2D) was performed according to Heng et al. [19] with some modifications. Powdered non-defatted solid sample (100 mg) was weighed into a 10 mL volumetric flask (*n* = 3), filled with aqueous EtOH (70%, *v*/*v*), mixed thoroughly, and ultrasonicated for 30 min (without additional heating). After ultrasonication, samples were centrifuged (14,000× *g* for 10 min at 10 °C) to remove insoluble matter. The supernatant (500 µL) was passed through PLD+ columns by applying positive pressure to remove proteins and phospholipids. The obtained filtrate was diluted to receive an aqueous MeCN (50%, *v*/*v*) solution. The diluted filtrate (100 µL) was transferred to the LC-MS vials, mixed with 50 µL soyasaponin Ba working solution and 50 µL U-^13^C-oat extract working solution, and injected into the LC-MS.

A homogeneous liquid sample was weighed (0.25 g) into a 5 mL volumetric flask (*n* = 3), filled with ultrapure water, and mixed thoroughly. Diluted sample solutions were centrifuged (14,000× *g* for 15 min at 10 °C) to remove insoluble matter. Sample supernatant (200 μL) and 800 µL MeCN were transferred into the next tube, mixed thoroughly, and centrifuged (14,000× *g* for 15 min at 10 °C) to remove precipitated proteins. The supernatant (500 µL) was passed through PLD+ columns. The obtained filtrate (300 μL) was transferred into a clear tube and diluted with 180 µL ultrapure water to obtain an aqueous MeCN solution (50%, *v*/*v*). The diluted sample filtrate was combined with internal standard solutions as described for solid samples and injected into the LC-MS.

### 2.4. Preparation of Standard Solutions

The stock solution of avenacoside A (500 mg/L) was prepared in ultrapure water and the aliquots were stored at −80 °C. The stock solution of saponin B (500 mg/L) was prepared in aqueous EtOH (60%, *v*/*v*) and aliquots were stored at −80 °C. The internal standard stock solution of soyasaponin Ba (100 mg/L) was prepared in MeOH.

The stock solution of U-^13^C-oat seed flour extract containing ^13^C_51_-avenacoside A was prepared using the previously described solid sample extraction method 2D with some modifications. U-^13^C-oat seed flour (150 mg) was weighed into a 50 mL volumetric flask, filled with EtOH (70%, *v*/*v*), and mixed thoroughly. The flask was ultrasonicated for 30 min (without additional heating) and the obtained solution was centrifuged (17,000× *g* for 10 min at 10 °C) to remove insoluble matter. The supernatant was passed through PLD+ columns using a vacuum manifold. The cleaned extract was aliquoted and stored at −80 °C.

The internal standard working solutions were prepared freshly before the analysis. The working solution of internal standard soyasaponin Ba was prepared by diluting stock solution in the aqueous MeCN (50%, *v*/*v*). The U-^13^C-oat extract working solution was prepared by diluting the stock solution two-fold with neat MeCN.

### 2.5. Liquid Chromatography Mass Spectrometry

Samples were analysed using a Waters UPLC^®^ system (Waters Corporation, Milford, MA, USA) coupled with a Waters Quattro Premier XE Mass Spectrometer equipped with ZSpray™ Source and controlled by Waters MassLynx™ 4.1 (V4.1 SCN805, Waters Corporation, Milford, MA, USA). Mobile phases were as follows: (A) 0.1% FA in ultrapure water, (B) 0.1% FA in MeCN. Weak needle wash was composed of MeCN in ultrapure water (90%, *v*/*v*), and strong needle wash consisted of IPA in MeCN (50%, *v*/*v*). The seal wash solution was aqueous MeCN (50%, *v*/*v*). Samples were stored in an autosampler which was set at 8 °C. The injection volume was 2 µL. Saponins were separated using BEH Amide column (1.0 × 50 mm, 1.7 μm) coupled with BEH Amide VanGuard Pre-column (2.1 × 5 mm) from Waters Corporation (Milford, MA, USA). The final gradient was as follows: 0–0.17 min at 10% A, 0.17–3.5 min linear gradient 10–70% A, 3.5–4.0 min at 70% A, 4.0–4.5 min linear gradient 70–10% A, 4.5–6.0 min at 10% A. The column temperature was held at 50 °C during all experiments. The flow rate was set at 200 µL/min.

The analytes were ionised under negative electrospray ionisation (ESI-) and optimised source conditions. The source temperature was set to 120 °C, and high-purity nitrogen was fed into the source at 25 L/h (cone) and 600 L/h (desolvation) and desolvation gas was heated to 350 °C. The capillary voltage was set to −1.5 kV, cone voltage to 80 V, and extractor voltage to 3 V. For measurement of analytes, a set of *m*/*z* values for single-ion-recording (SIR) experiments was recorded simultaneously during one chromatographic run. For saponin quantification, deprotonated molecules [M-H]- were chosen based on a scan-type experiment. Mass-to-charge ratios (*m*/*z* ± 0.5 Da) for SIR channels were set as follows: avenacoside A—*m*/*z* 1061.5; avenacoside B—*m*/*z* 1223; 26-desglucoavenacoside A—*m*/*z* 899.5; ^13^C_51_-avenacoside A—*m*/*z* 1112.5 (internal standard); saponin B—*m*/*z* 941.5; DDMP saponin—*m*/*z* 1067; soyasaponin Ba—*m*/*z* 957.5 (internal standard). Data acquisition was performed in Waters MassLynx™ V4.1 (SCN805, Waters Corporation, Milford, MA, USA). Data analysis was performed in Waters QuanLynx™ V4.1 (SCN805, Waters Corporation, Milford, MA, USA) and Microsoft Excel^®^ (Microsoft 365 Apps for enterprise).

### 2.6. Calibration and Quantification

The working solution was prepared by diluting standard stock solutions 100 times with MeCN:H_2_O:EtOH solution (50:36:14, *v*/*v*). Internal standards, soyasaponin Ba and U-^13^C-oat extract, were added before injection to the autosampler vial, and their concentration in the vial was set at 0.75 mg/L and 0.3 mg/L, respectively. Calibration curve standard solutions (100 µL) were mixed with internal standards working solutions (50 µL U-^13^C-oat extract working solution and 50 µL soyasaponin Ba working solution). Calibration curves were built for avenacoside A (0.01–2.44 mg/L) and saponin B (0.01–2.48 mg/L) using eight-point measurements of serially diluted standards, which were run in triplicate. The regression was found by fitting points to the linear equation. The external standard calibration curves were built by correlating the concentrations of external standards to the response factors, which were calculated according to Equation (1).

response factor (RF) = (area of analyte)/(area of internal standard)(1)

As only the avenacoside A standard was commercially available, other analytes of interest (avenacoside B and 26-desglucoavenacoside A) were quantified relatively using the avenacoside A calibration curve. Avenacoside B and 26-desglucoavenacoside A results are presented in avenacoside A equivalents. Avenacosides were quantified using isotopically labelled ^13^C-avenacoside A as an internal standard. As DDMP saponin could not be sourced commercially, its quantification was based on the saponin B standard curve, and the results are given in saponin B equivalents. Both were quantified using soyasaponin Ba as an internal standard.

### 2.7. Validation of the Method

The following parameters were assessed during method validation: linearity, limit of detection (LOD), limit of quantification (LOQ), precision, specificity, sample extraction recoveries, and matrix effect (ME). Developed extraction methods for solid and liquid samples were validated separately. Oat protein concentrate and pea protein isolate were used to validate the solid sample extraction method. Saponin determination in liquid samples was validated using oat and pea drinks.

The linear range and linearity were evaluated via repeated measurements of standard solutions of avenacoside A and saponin B consisting of 8 individual points obtained from serial dilution of stock solutions. For the calculation of LOD and LOQ values for avenacoside A and saponin B compounds, the standard deviation (SD), obtained by analysing the peak areas of the lowest standard concentration point, was multiplied by three or ten, respectively [42].

To determine the intra-day precision of the instrumental method, oat protein concentrate and pea protein isolate extracts containing all analytes and internal standards were injected six times, and for inter-day precision, sample extracts were studied across three independent days to confirm the stability of the retention times and peak areas. The precision of the extraction methods was determined by repeatability (intra-day) and intermediate precision (inter-day). Repeatability was carried out by performing six repeated analyses of the samples on the same day, while the intermediate precision of the method was assessed using samples that were analysed on three different days over two months under the same experimental conditions.

The total recoveries for avenacoside A and saponin B were evaluated by spiking the solid and liquid samples with a known amount of avenacoside A and saponin B at three different concentration levels. For estimation of solid sample extraction method recovery, oat protein concentrate and pea protein isolate (100 mg) were weighed into a 10 mL volumetric flask (*n* = 3). Aliquots of avenacoside A and saponin B standard solutions (10 mL) at three different concentrations were prepared in aqueous EtOH (70%, *v*/*v*) separately. These solutions were added to oat protein concentrate and pea protein isolate, mixed thoroughly and subjected to the solid sample extraction method as described above. The recoveries of avenacoside A and saponin B in oat and pea liquid samples were determined by cross-matrix spiking both sample matrices. For estimation of liquid sample extraction method recovery, separate standard stock solutions of avenacoside A and saponin B were prepared (200 mg/L). These solutions were added in different volumes to 0.25 g of liquid sample (oat and pea drink) (*n* = 3) weighed into a 5 mL volumetric flask, mixed thoroughly, and subjected to the liquid sample extraction method as described above. The total recovery was calculated using Equation (2) [43],

total recovery (%) = (C_spiked_/(C_unspiked_ + C_spike_)) × 100% (2)

where C_spiked_ is the amount of saponin determined in the spiked sample, C_unspiked_ is the amount of saponin in the unspiked sample, and C_spike_ is the amount of saponins at three different concentration levels.

ME as one of the most problematic issues in LC-MS was evaluated for all four sample matrices (oat protein concentrate and pea protein isolate and plant-based drinks) by post-extraction sample spiking with calibration curve standard solutions, then constructing a calibration curve based on response factors and spiked standard concentrations, and comparing the matrix-matched calibration curve slope with the calibration curve slope in solvent (Equation (3)) [42]

ME (%) = slope_matrix-matched_/slope_solvent_ × 100%. (3)

Statistical analysis was carried out using Excel^®^ (Microsoft^®^ 365 for enterprise). The results are presented as mean ± SD or relative standard deviation (RSD).

## 3. Results and Discussion

### 3.1. Development of Liquid Chromatography Method

The HPLC method was developed and assessed by analysing external standards and compounds available in oat and pea sample matrices. During development of the liquid chromatography method, two types of stationary phase chemistry were tested (C18 and HILIC) as well as different column dimensions. The best separation performance in terms of time of analysis, selectivity, and efficiency was achieved by the BEH Amide column (1.0 × 50 mm, 1.7 μm). Based on the literature [9,19] and scan-type experiments of oat flour and pea flour sample extracts, *m*/*z* values for SIR channels were chosen for the detection and relative quantification of targeted compounds without existing standard compounds in these sample matrices. Avenacoside B and 26-desglucoavenacoside A were found to be present in the oat sample matrix in addition to avenacoside A. DDMP saponin also occurred in the pea sample matrix besides saponin B. MRM experiments were conducted during development of a methodology but we have found that the MRM approach did not bring any more selectivity but significantly reduced sensitivity by not producing consistent fragments. The example of a chromatogram obtained by injecting the oat and pea flour extracts is shown in Supplementary Figure S1.

### 3.2. Development of Sample Extraction Methods

Two previously published extraction methods (avenacosides in grain and husks of oats [9] and saponins in peas [19]) were the starting points for the development of a method for simultaneous saponin extraction from oat and pea matrices. As both extraction methods were time-consuming, a more efficient sample preparation was proposed for saponin quantification. All samples were analysed using LC-MS method described in the Materials and Methods section.

Table 1 shows the main steps of extraction methods and saponin extraction yields obtained by reference methods (1A and 2A) and modified methods (1B, 2B, 2C, and 2D). To demonstrate the efficiency of the optimized methods, oat protein concentrate and pea protein isolate were analysed in duplicate.

Since both reference methods [9,19] started by fat elimination, defatted oat protein concentrate (fat 18.9%) and pea protein isolate (fat 4.7%) were extracted using methods 1A, 1B, 2A, and 2B. The oat protein concentrate extracted using method 1B gave 37% higher avenacoside A concentration compared to method 1A, and method 2B resulted in 50% higher yield than method 2A. Overall, the highest avenacoside A content in oat protein concentrate was achieved using extraction method 2B. Using method 1B, the pea protein isolate gave two times higher saponin B yield than using extraction method 1A, and method 2B gave a 76% higher yield than method 2A. Thus, the highest saponin B amount from pea protein isolate was extracted using method 2B. Although both improved methods 1B and 2B gave similar saponin yields in analysed matrices, it was decided to proceed with more process-efficient method 2B, as method 1B utilizing two-step methanol reflux extraction is very time-consuming.

The necessity for fat removal before saponin extraction from the matrix was determined. For this, saponins from four samples (oat flour and protein concentrate and pea flour and protein isolate) were extracted using extraction methods 2B and 2C, and lastly, the extracts were filtered through different filtering devices (the molecular weight cut-off filters with different membrane pore sizes (3, 10, 30, and 50 kDa), 0.2 μm syringe filter, and ISOLUTE^®^ PLD+ Protein and Phospholipid Removal columns) before the LC-MS analysis. The results of this experiment are shown in Supplementary Figure S2.

No significant differences in avenacoside A, avenacoside B, saponin B, and DDMP saponin content were determined in Soxhlet-defatted and non-defatted oat and pea matrices. On the other hand, different molecular cut-off sizes had a significant impact on the recovery of saponins. The 3 kDa and 10 kDa cut-off filters showed inferior performance irrespective of the sample matrix and saponin type determined. The maximum recovery of analytes in the samples was achieved using 50 kDa and in some cases 30 kDa cut-off devices. In all sample matrices except oat protein concentrate, the application of PLD+ columns and syringe filters gave even better results than 30 kDa or 50 kDa cut-off filters. Although the PLD+ and 0.2 μm syringe filters gave quite similar analyte recovery, the application of PLD+ columns resulted in clearer MS chromatograms with a minimum number of interfering peaks in the chromatogram baseline. Moreover, filtering through the PLD+ column enables an easy transition of the procedure to a high-throughput workflow in the case of using 96-well PLD+ plates. The ISOLUTE^®^ PLD+ proprietary multifunctional sorbent phase is optimised to selectively retain proteins and phospholipids [44]. The results indicated that pre-extraction fat removal is not necessary before saponin extraction and could be omitted and the application of PLD+ columns is the best solution for post-extraction clean-up of sample extracts. This resulted in a modified method 2C (described in Table 1).

The influence of ultrasonic power on the saponin extraction yields was also investigated. Saponins from oat protein concentrate, pea protein isolate, and oat and pea flours were extracted using methods 2C and 2D (results are shown in Supplementary Table S2). The results showed that ultrasonication did not have a statistically significant effect on saponin yield but considering the extraction time the application of ultrasonication is preferable. It should be noted that heating taking place during sonication had no effect on the analytes. During this experiment, the ultrasonic bath heated itself from ambient temperature (23 °C) to 40 °C in 30 min. Previous research has shown that the exposure of DDMP saponin to a temperature higher than 40 °C has a profound effect on its degradation into saponin B [18]. However, in another study, it was reported that the pure DDMP saponin in methanolic solution started to decrease in concentration when heated at 65 °C [45].

Based on the obtained results and considering the extraction time and yield, method 2D was utilized for analysis and validation of all solid samples.

Liquid food samples were analysed without the need to use lyophilisation before the sample extraction. The sample preparation method was based only on the application of ISOLUTE^®^ PLD+ cartridges for sample extract purification before LC-MS analysis, previously chosen as the most efficient for cleaning the extracts of the solid samples.

### 3.3. Validation of the Method

When the chromatographic methods and sample extraction methods were developed, validation was performed to evaluate the linear range, LODs and LOQs, precision, recoveries, and matrix effect of the proposed method. The linearity of response and other calibration parameters for avenacoside A and saponin B are presented in Table 2. Linearity for these two saponin standards was obtained in the concentration range of 0.01–2.5 mg/L. The LOQs were estimated from the lowest point of the calibration curve ranging from 0.015 mg/L for avenacoside A and 0.014 mg/L for saponin B. The obtained LOQ results were lower than or in accordance with previous research [9,13,17,39].

After linearity was found to be acceptable for avenacoside A and saponin B, the repeatability of the method was appraised. Repeatability of retention times and peak areas were studied first with six replicate injections of oat protein concentrate and pea protein isolate extract. Table 3 shows the repeatability of retention times, peak areas, and the precision of solid and liquid sample extraction methods. RSDs of peak areas for all saponins did not exceed 6%. Intra- and inter-day RSDs were at a similar level, indicating that the methods are reproducible to an acceptable extent for the routine analysis of oat and pea products. Intra-day and inter-day RSDs were determined by extracting oat protein concentrate, pea protein isolate, and plant-based drinks on different days. The RSD of the intra-day precision ranged from 6 to 13% and inter-day precision from 7 to 11% in powdered oat and pea samples. For oat and pea plant-based drinks, the intra-day precision ranged from 3 to 12% and inter-day precision from 7 to 16%. The precisions for the DDMP saponin pea drink were not evaluable despite multiple measurements (DDMP saponin content in this sample was <LOQ).

The recoveries were determined in oat protein concentrate and pea protein isolate powder by spiking the oat matrix with avenacoside A and the pea matrix with saponin B. The recovery of analytes in the case of the liquid sample extraction method was investigated separately. Table 4 shows the recovery results of powdered and liquid samples. The recoveries of avenacoside A and saponin B ranged from 90 to 115% and from 82 to 100% in oat protein concentrate and pea protein isolate, respectively. In the oat drink, the recovery of avenacoside A ranged from 96 to 113% and saponin B from 98 to 113%. In the pea drink matrix, the recoveries of avenacoside A and saponin B were from 94 to 106% and from 89 to 98%, respectively. According to validation guidelines, the acceptable recovery range for this method should be in the range of 80 to 110% [46]. Thus, the mean values of obtained recoveries were acceptable for both matrices. The recovery results obtained with the current procedure were similar to ones reported for previously proposed methods [9,13].

Oat protein concentrate ME on avenacoside A was 100%, and pea protein isolate ME on saponin B was 110%. Avenacoside A and saponin B ME were 107% and 105% in the oat drink and 105% and 102% in the pea drink, respectively. All measured ME were in the optimal range between 90 and 110% [47].

The stock solution of U-^13^C-oat seed flour extract was analysed for purity. The unlabelled avenacosides were not detected; thus, isotopically labelled avenacoside A was regarded as fully labelled. The working solution of ^13^C-oat flour was added into the LC-MS vial before the analysis to assess the quantity of analytes and take into account ME. Moreover, recovery experiments confirmed that the method could be used even with internal standards added post-extraction.

Overall, the method has demonstrated acceptable validation performance in terms of recovery, sensitivity, specificity, and precision, and could be characterised as robust and effective and could potentially be applied in a high-throughput environment. Thus, the developed sample extraction method and the LC-MS method are suitable tools for the analysis of oat and pea saponins in different matrices, e.g., flours, protein concentrates and isolates, mixed matrices, and liquid plant-based drinks.

### 3.4. Determined Concentrations of Saponins in Food Ingredients, Half- and End-Products

High sensitivity and reproducibility as well as very short analysis time make the developed method suitable for routine quality analysis of oat- and pea-based food ingredients and foods, as well as products containing oat and pea components. The results of saponin contents in various samples are shown in Table 5.

In whole-grain oat flour, the contents of avenacoside A, avenacoside B, and 26-desglucoavenacoside A were 23.4 ± 2.9 mg/100 g, 14.0 ± 1.5 mg/100 g, and below LOQ, respectively. According to previous research, the concentrations of avenacosides and their ratios are different and depend largely on the variety of oats [9]. According to the latter study, the average avenacoside A content in oat grain in 16 analysed varieties was 36 ± 8 mg/100 g, avenacoside B content was in the range of 30 ± 4 mg/100 g, and 26-desglucoavenacoside A was 2.4 ± 0.8 mg/100 g [9]. Indeed, the contents of avenacoside A differed up to two-fold depending on the variety, and the ratios of avenacoside A to avenacoside B varied from 0.9 to 1.7 [9]. According to Günther-Jordanland et al. (2020), avenacoside A and avenacoside B content in oat flour has been reported to be 24.6 mg/100 g and 21.9 mg/100 g, respectively [13]. Thus, the concentration of avenacosides in the whole-grain oat flour determined in the present study is in a good correspondence with the results reported before [9,13]. In oat protein concentrate (53% protein; Table S1), avenacoside A content was 42.3 ± 3.0 mg/100 g, avenacoside B was 33.8 ± 0.7 mg/100 g, and 26-desglucoavenacoside A was 5.1 ± 0.2 mg/100 g. According to specification (Table S1), this product was manufactured from oat bran. Previous research has shown that the average content of avenacoside A and avenacoside B in three analysed oat bran products was 26 ± 7 mg/100 g and 8 ± 2 mg/100 g, respectively [17], which is similar to concentrations determined in the whole-grain flour in the current study. Thus, the increased content of avenacosides in oat protein concentrate should be ascribed to the partial concentration of the oat saponins together with the protein fraction during the production process of oat protein concentrate. In an oat drink, avenacoside A content was 4.6 ± 0.1 mg/100 g, avenacoside B was 2.7 ± 0.2 mg/100 g, and 26-desglucoavenacoside A was below LOQ. As it was a commercial liquid product with low dry matter content, it resulted in an apparently lower content of measured saponins. Nevertheless, according to specification (Table S1), the product contains only 1% of protein and the oat base is the only protein source in the oat drink. In this respect, considering the oat drink and, e.g., the whole-grain oat flour (12.5% of protein), the ratio of avenacosides to protein is much higher in the oat drink. One can suppose the considerable migration of saponins into the liquid phase when soaking the oats during the initial step of oat drink manufacture.

In pea flour (17.9% protein; Table S1), saponin B content was 6.2 ± 0.4 mg/100 g and relatively quantified DDMP saponin content was 61.1 ± 2.0 mg/100 g. In fact, our findings are inconsistent with the results of Reim and Rohn (2015), who analysed saponin B and DDMP saponin contents in hulls and peas in six different pea varieties using the HPTLC method [32]. They reported that saponin content in peeled peas was 10 to 40 mg/100 g of saponin B and 0 to 20 mg/100 g of DDMP saponin depending on pea variety [32]. Nonetheless, the present findings of high DDMP content in pea flour are comparable with the results of Heng et al. (2006): the DDMP saponin content varied from 70 to 150 mg/100 g DM, whereas saponin B varied from 0 to 40 mg/100 g DM [19]. Our results confirm that the DDMP saponin is the predominant naturally occurring saponin present in pea. The high level of DDMP saponin in pea flour was observed in the current study most likely because it has not been thermally treated and DDMP saponin has not been converted into saponin B. In pea protein concentrate (46.9% protein; Table S1), the saponin B content was 80.3 ± 1.6 mg/100 g and DDMP content was 107.6 ± 4.1 mg/100 g. Saponins are found in the cotyledons and are often associated with the protein bodies of legumes [4]. Therefore, saponin accumulation in pea concentrate produced by dry milling and air classification is evident [4], which is in accordance with at least twice higher levels of saponins in pea protein concentrate compared to pea flour determined in our study. In pea protein isolate (75% protein; Table S1), saponin B content was 243.8 ± 6.2 mg/100 g and DDMP content was 10.8 ± 0.7 mg/100 g. These results show that protein wet extraction and isoelectric precipitation, likely performed to achieve protein isolate, degrade unstable DDMP saponin naturally occurring in peas into saponin B. In the pea drink, saponin B content was 3.5 ± 0.2 mg/100 g and DDMP saponin was below LOQ. According to the product specification (Table S1), it contains 2% of protein, and the only protein source is pea. Although the exact production process of the pea drink is unknown, taking into account the content of saponin B per 1 g of pea drink protein (1.75 mg), the probable pea protein source should contain at least 175 mg of saponins (sum of saponin B and DDMP saponin, as DDMP saponin is converted into saponin B during drink pasteurization) per 100 g of pure pea protein.

To test the applicability of the developed method for simultaneous determination of oat and pea saponins from one matrix, the blend of pea isolate, oat protein concentrate, and pea protein concentrate was used. In addition, the part of the mixture was extruded according to the previously published article [41]. Results show that avenacoside A, avenacoside B, 26-desglucoavenacoside A, saponin B, and DDMP saponin content in the blend were 13.5 ± 1.0 mg/100 g, 10.9 ± 0.3 mg/100 g, 1.3 ± 0.3 mg/100 g, 123.9 ± 6.2 mg/100 g, and 27.1 ± 3.5 mg/100 g, respectively. Considering that this blend was composed of 52% pea protein isolate, 28% oat protein concentrate, and 20% pea protein concentrate, which were also analysed separately, the recoveries of avenacoside A, avenacoside B, 26-desglucoavenacoside A, saponin B, and DDMP saponin were 114%, 115%, 90%, 95%, and 100%, respectively. In the extruded blend, avenacoside B and 26-desglucoavenacoside A content did not change significantly, avenacoside A content decreased by 21%, and saponin B content increased from 123.9 to 132.9 mg/100 g, which could potentially happen due to DDMP saponin conversion into saponin B during extrusion cooking.

## 4. Conclusions

In conclusion, the HILIC-MS-based method for oat and pea matrices, with a relatively simple extraction procedure for solid and liquid samples, allowing the simultaneous quantification of avenacoside A and saponin B, and the relative quantification of avenacoside B, 26-desglucoavenacoside A, and DDMP saponin, was employed for analysis of saponins in various food ingredients and products. Oat protein concentrate, pea protein isolate, and oat- and pea-based drinks were chosen for development and validation of the sample extraction methods. The optimised HILIC-MS method was able to absolutely quantify avenacoside A and saponin B in the matrices; other compounds were quantified based on existing standard compounds. The validation of the improved methods for both sample types (solid and liquid) showed the acceptable linear range, LODs and LOQs, precisions, recoveries, and MEs. Generally, an inter-day precision was below 20%. The accuracy and the precision of quantification were achieved by using the labelled internal standard (^13^C-avenacoside A) obtained from U-^13^C-labelled oat flour and with soyasaponin Ba as internal standards. The content of saponins was measured in different plant-based oat and pea products (ingredients, half- and end-products). This method could be potentially extended for other plant-based sample matrices, and the absolute quantification of all analytes could be achieved if the missing saponin standards were to arrive on the market.

## Figures and Tables

**Table 1:** The extraction steps of reference methods 1A and 2A [9,19], their modified versions (1B, 2B, 2C, and 2D), and saponin yields obtained using these methods ^a^.

	Sample Extraction 1A	Modified Sample Extraction 1B	Sample Extraction 2A	Modified Sample Extraction 2B	Modified Sample Extraction 2C	Modified Sample Extraction 2D
Defatted sample	yes	yes	yes	yes	no	no
Sample and solvent amount (10 g/L)	0.5 g, 25 mL × 2 MeOH	0.5 g, 25 mL × 2 MeOH	0.5 g, 50 mL EtOH (70%, *v*/*v*)	0.1 g, 10 mL EtOH (70%, *v*/*v*)	0.1 g, 10 mL EtOH (70%, *v*/*v*)	0.1 g, 10 mL EtOH (70%, *v*/*v*)
Extraction	2-step reflux at boiling point	2-step reflux at boiling point	1 h at 25 °C in a shaking incubator	1 h at 25 °C in a shaking incubator	1 h at 25 °C in a shaking incubator	30 min ultrasonic bath
Clean-up	decant	centrifuge (17,000× *g* × 10 min at 10 °C)	ashless filter paper	centrifuge (17,000× *g* × 10 min at 10 °C)	centrifuge (14,000× *g* × 10 min at 10 °C)	centrifuge (14,000× *g* × 10 min at 10 °C)
Solvent evaporation	vacuum rotary evaporator	-	vacuum rotary evaporator	-	-	-
Resuspended	an aqueous MeOH (5%, *v*/*v*)	-	-	-	-	-
Centrifuge	17,000× *g* × 10 min at 10 °C	-	17,000× *g* × 10 min at 10 °C	-	-	-
Sample clean-up and concentration	SPE C18	-	SPE C18	-	-	-
Solvent evaporation	N_2_ flow	-	N_2_ flow	-	-	-
Filtering	-	-	-	-	PLD+ column	PLD+ column
Reconstitution/dilution in an aqueous MeCN (50%, *v*/*v*)	yes	yes	yes	yes	yes	yes
Obtained results (mg/100 g ± SD)						
Avenacoside A ^b^	19 ± 2 ^c^	26 ± 1 ^c^	18 ± 3 ^c^	37 ± 2 ^c^	36 ± 3	37 ± 2
Saponin B ^d^	100 ± 3 ^c^	214 ± 5 ^c^	124 ± 10 ^c^	219 ± 8 ^c^	229 ± 22	240 ± 19

^a^ Detailed description of extraction methods 1A, 1B, 2A, 2B, and 2C is available in the supplementary information. Each result represents mean ± SD (*n* = 2). ^b^ Measured in oat protein concentrate. ^c^ Result is presented on fat-containing sample. ^d^ Measured in pea protein isolate.

**Table 2:** The linear range, calibration curve, limits of detection (LODs), and limits of quantification (LOQs) of avenacoside A and saponin B.

Analyte	Linear Range (mg/L)	Calibration Curve ^1^	R^2^	LOD (mg/L)	LOQ (mg/L)
avenacoside A	0.01–2.44	y = 0.2445x − 0.0197	0.9998	0.004	0.015
saponin B	0.01–2.48	y = 0.6350x − 0.0059	0.9999	0.004	0.014

^1^ Calibration curve: y = ax + b, where x is the response factor and y is the concentration in mg/L.

**Table 3:** Repeatability of retention times (RT) and peak areas of saponins, and precision of the whole method.

Analyte	*m*/*z*	RT, min	RT RSD (%)		Peak Area RSD (%)		Precision RSD (%)			
			Intra-Day (*n* = 6)	Inter-Day (*n* = 18)	Intra-day (*n* = 6)	Inter-Day (*n* = 18)	Powdered Samples		Plant-Based Drinks	
							Intra-Day (*n* = 6)	Inter-Day (*n* = 9)	Intra-Day (*n* = 6)	Inter-Day (*n* = 9)
Avenacoside A	1061.5	1.78	0.23 ^a^	0.48 ^a^	1.8 ^a^	3.0 ^a^	11	11	12	12
Avenacoside B	1223	1.93	0.20 ^a^	0.27 ^a^	4.1 ^a^	4.5 ^a^	13	9	3	8
26-desglucoavenacoside A	899.5	1.54	0.36 ^a^	0.56 ^a^	3.8 ^a^	6.0 ^a^	6	7	10	16
^13^C-avenacoside A ^b^	1112.5	1.78	0.28	0.54	2.9	4.2	n.i.	n.i.	n.i.	n.i.
Saponin B	941.5	1.42	0.29 ^c^	0.98 ^c^	2.6 ^c^	3.1 ^c^	6	7	6	7
DDMP saponin	1067	1.40	0.23 ^c^	0.86 ^c^	3.9 ^c^	6.3 ^c^	8	11	n.a.	n.a.
Soyasaponin Ba ^d^	957.5	1.48	0.30	0.68	2.5	3.0	n.i.	n.i.	n.i.	n.i.

^a^ Measured in oat protein concentrate. ^b^ Internal standard for avenacoside A, avenacoside B, and 26-desglucoavenacoside A. n.i. Not implemented, no RSD calculable. ^c^ Measured in pea protein isolate. n.a. Not available in this matrix. ^d^ Internal standard for saponin B and DDMP saponin.

**Table 4:** Recoveries of saponins in solid (oat protein concentrate and pea protein isolate) and liquid samples (oat drink and pea drink) ^1^.

	Spiking Level	Spike (mg/L)	Recovery (%)	Spike (mg/L)	Recovery (%)	Recovery (%)
			Oat Protein Concentrate ^2^		Oat Drink ^3^	Pea Drink ^4^
Avenacoside A	L1	1.2	115 ± 7	0.24	104 ± 6	106 ± 1
	L2	0.9	90 ± 7	0.12	113 ± 7	99 ± 3
	L3	0.4	107 ± 12	0.06	96 ± 13	94 ± 1
			Pea protein isolate ^5^		Oat drink ^4^	Pea drink ^6^
Saponin B	L1	1.1	82 ± 2	0.23	105 ± 2	98 ± 5
	L2	0.5	100 ± 1	0.11	113 ± 8	89 ± 2
	L3	0.3	96 ± 10	0.06	98 ± 15	93 ± 6

^1^ Each result represents mean ± SD (*n* = 3). ^2^ Unspiked matrix initial analyte concentration 0.70 mg/L. ^3^ Unspiked matrix initial analyte concentration 0.45 mg/L. ^4^ Analyte-free sample matrix. ^5^ Unspiked matrix initial analyte concentration 0.55 mg/L. ^6^ Unspiked matrix initial analyte concentration 0.15 mg/L.

**Table 5:** Contents of saponins in different food samples ^1^.

	mg/100 g				
Sample	Avenacoside A	Avenacoside B ^2^	26-desglucoavenacoside A ^2^	Saponin B	DDMP Saponin ^3^
Oat protein concentrate	42.3 ± 3.0	33.8 ± 0.7	5.1 ± 0.2	n.a.	n.a.
Whole-grain oat flour	23.4 ± 2.9	14.0 ± 1.5	<LOQ	n.a.	n.a.
Oat drink	4.6 ± 0.1	2.7 ± 0.2	<LOQ	n.a.	n.a.
Pea protein isolate	n.a.	n.a.	n.a.	243.8 ± 6.2	10.8 ± 0.7
Pea protein concentrate	n.a.	n.a.	n.a.	20.3 ± 1.6	107.6 ± 4.1
Pea flour	n.a.	n.a.	n.a.	6.2 ± 0.4	61.1 ± 2.0
Pea drink	n.a.	n.a.	n.a.	3.5 ± 0.2	<LOQ
Blend ^4^	13.5 ± 1.0	10.9 ± 0.3	1.3 ± 0.3	123.9 ± 6.3	27.1 ± 3.5
Extruded blend ^4^	10.6 ± 0.3	9.6 ± 0.9	1.1 ± 0.9	132.9 ± 12.4	11.4 ± 0.8

^1^ Each result represents mean ± SD (*n* = 3). ^2^ Equivalent of avenacoside A. ^3^ Equivalent of saponin B. n.a. Not available in this sample matrix. ^4^ Blend: 52% pea protein isolate, 28% oat protein concentrate, and 20% pea protein concentrate.

## Data Availability

Data is contained within the article or supplementary material.

## Supplementary Materials

The following supporting information can be downloaded at https://www.mdpi.com/article/10.3390/foods12050991/s1. Description of solid sample extraction methods (1A, 1B, 2A, 2B, and 2C) used during the method development; Table S1: Nutritional information of analysed products; Figure S1: LC-MS chromatograms of oat and pea flours (SIR and ESI-). In oat flour: avenacoside A, avenacoside B, 26-desglucoavenacoside A, and internal standard ^13^C-avenacoside A. In pea flour: saponin B, DDMP saponin, and internal standard soyasaponin Ba; Figure S2: Saponin yield in (a) oat and (b) pea matrices. The effect of sample clean-up: the pre-extraction of fat and six post-extraction filtration possibilities. The results of avenacoside B are presented in equivalents of avenacoside A mg/g and DDMP saponin in equivalents of saponin B mg/g; Table S2: The effectiveness of ultrasonic bath extraction compared to reference extraction conditions using the tube rotator (extraction yield 100%).
