# Supplementary material for: Quantitative Analysis of Oat (Avena sativa L.) and Pea (Pisum sativum L.) Saponins in Plant-Based Food Products by Hydrophilic Interaction Liquid Chromatography Coupled with Mass Spectrometry
Source: Foods. 2023 Feb 26;12(5):991. doi: 10.3390/foods12050991 (PMC10000715; doi:10.3390/foods12050991)
Supplement: Supplementary file 1 [file foods-12-00991-s001.zip › foods-2224971-supplementary.pdf]

Supplementary Information

Quantitative Analysis of Oat (*Avena sativa* L.) and Pea (*Pisum sativum* L.) Saponins in Plant-Based Food Products by Hydrophilic Interaction Liquid Chromatography Coupled with Mass Spectrometry

Anastassia Bljahhina[1,2,*], Dmitri Pismennõi[1,2], Tiina Kriščiunaite[1], Maria Kuhtinskaja[2] and Eeva-Gerda Kobrin[1]

[1]Center of Food and Fermentation Technologies (TFTAK), Mäealuse 2/4, 12618 Tallinn, Estonia

[2]Department of Chemistry and Biotechnology, Tallinn University of Technology, Akadeemia Tee 15, 12618 Tallinn, Estonia

* Correspondence: anastassia.bljahhina@tftak.eu

Description of solid sample extraction methods (1A, 1B, 2A, 2B and 2C) used during the method development

Sample extraction method 1A

Solid sample extraction was performed according to literature (Pecio et al. 2013). Samples were defatted using Ser158 Solvent auto extractor (VELP Scientifica Srl, Italy). For that, sample was weighed (6 g) into extraction thimbles. The extraction was carried out with 100 mL hexane using auto extractor program parameters based on the operation manual of auto extractor. Defatted samples were used later in development of the solid sample extraction method. Reflux equipment was set up as described previously (Pecio et al. 2013). Defatted sample (0.5 g) were extracted twice using 25 mL of MeOH during 1 h at boiling point under reflux. Solvents were combined and evaporated under vacuum on rotary evaporator. Residue was resuspended in an aqueous MeOH (5%, v/v). Solid phase extraction C18 column was used to concentrate analytes and clean the sample extract. After solid phase extraction step sample was reconstituted in an aqueous MeCN (50%, v/v) before injecting into LC-MS.

Sample extraction method 1B

Solid sample extraction was performed according to literature with modifications (Pecio et al. 2013). Samples were defatted and extracted as described in method 1A, but after 2-step reflux extracts were combined and centrifuged (17,000 *g* x 10 min at 10 °C) and diluted sample extract [achieve MeCN (50%, v/v)] was injected to LC-MS.

Sample extraction method 2A

Solid sample extraction was performed according to literature (Heng et al. 2006). Samples were defatted as described in method 1A. Defatted sample (0.5 g) was extracted with EtOH (50 ml, 70%, v/v) during 1 h in shaking incubator. Sample extract was filtered through ashless filter paper and EtOH was evaporated from the extract. The resulting turbid solution was centrifuged (17,000 *g* x 10 min at 10 °C). Solid phase extraction C18 column was used to concentrate analytes and clean the sample extract. After solid phase extraction step sample was reconstituted in an aqueous MeCN (50%, v/v) before injecting into LC-MS.

Sample extraction method 2B

Solid sample extraction was performed according to literature with modifications (Heng et al. 2006). Samples were defatted and extracted as described in method 1A, but after EtOH extraction sample extract was centrifuged (17,000 *g* x 10 min at 10 °C) and diluted sample extract [achieve MeCN (50%, v/v)] was injected to LC-MS.

Sample extraction method 2C

Solid sample extraction was performed according to method 2B with modifications. Non-defatted samples were used in this extraction method. After EtOH extraction (described in method 2B), samples were centrifuged and submitted to Biotage Isolute PLD+ cartridges to remove proteins and phospholipids. Before injecting sample to LC-MS sample filtrate was diluted with MeCN to achieve 50% solution.

Table S1 Nutritional information of analysed products

**product**	**information**
**yellow pea flour**	Nutritional values per 100g: Energy 1250 kJ/ 299 kcal, Fat 1g of which saturated 0,2g, Carbohydrates 59.2g of which sugars 1.8g, Protein 17.9g, Salt 4mg, Dietary fibre 10.7g.
**whole grain oat flour**	Nutritional values per 100g: Energy 1500kJ/357 kcal, Fat 6.5g of which saturated 1.2g, Carbohydrates 57g of which sugars 0.7g, Protein 12.5g, Salt 1mg.
**pea drink**	Ingredients: Water, Rapeseed Oil, Pea Protein (2.5%), Sugar, Acidity Regulator: Dipotassium Phosphate, Carriers: (Calcium Carbonate, Calcium Phosphate), Gluten-Free Oat Oil, Salt, Vitamins (D3, Riboflavin and B12)<br>Nutritional values per 100ml: Energy: 176 kJ / 43 Kcal Fat: 3.0g of which saturated: 0.3g Carbohydrates: 1.8gof which sugars: 1.8g Protein: 2.0g Salt: 0.2g Vitamin D: 1.0 microgram, Riboflavin: 0.21 micrograms, Vitamin B12: 0.38 micrograms Calcium: 120mg
**oat drink**	Ingredients: Oat base (water, oats 10%), Rapeseed oil, acidity regulator: Dipotassium phosphate, Carriers: (Calcium Carbonate, Calcium Phosphate), Salt, Vitamin (D3, Riboflavin and B12)<br>Nutritional values per 100 ml: Energy 256 kJ / 62 Kcal, Fat 2.8 g, Carbohydrates 7.8 g of which sugars 3.5 g, Protein 1 g, Salt 0.1 g Vitamin D 1.0 micrograms, Riboflavin 0.21 micrograms, Vitamin B12 0.38 micrograms, Calcium 120mg
**pea protein concentrate**	Ingredients: Organic peas. Dehulled, ground and air classified pea.<br>Energy and nutritional value 100 g: Energy value (kJ/kcal) 1387 kJ - 331 kcal, Fat 3,4g of which: saturates 0,6 g, Carbohydrates 34,8g of which: sugars 6,2g, Fibres 13,2 g, Protein 46,9g, Salt 0,01g
**pea protein isolate**	Nutritional values per 100g: energy 1680 kJ/ 398 kcal, fat 3.32g, carbohydrates 1.54g, proteins 74.9g
**oat protein concentrate**	Raw material: oat bran<br>Nutritional values per 100g: energy 1770 kJ/ 423 kcal, fat 13g of which saturated 2.6g, monounsaturated fat 4.2g, polyunsaturated fat 5.6g, carbohydrates 21g of which sugars 0.7g, fibres 4.6g, Protein 53g, salt 0.11g

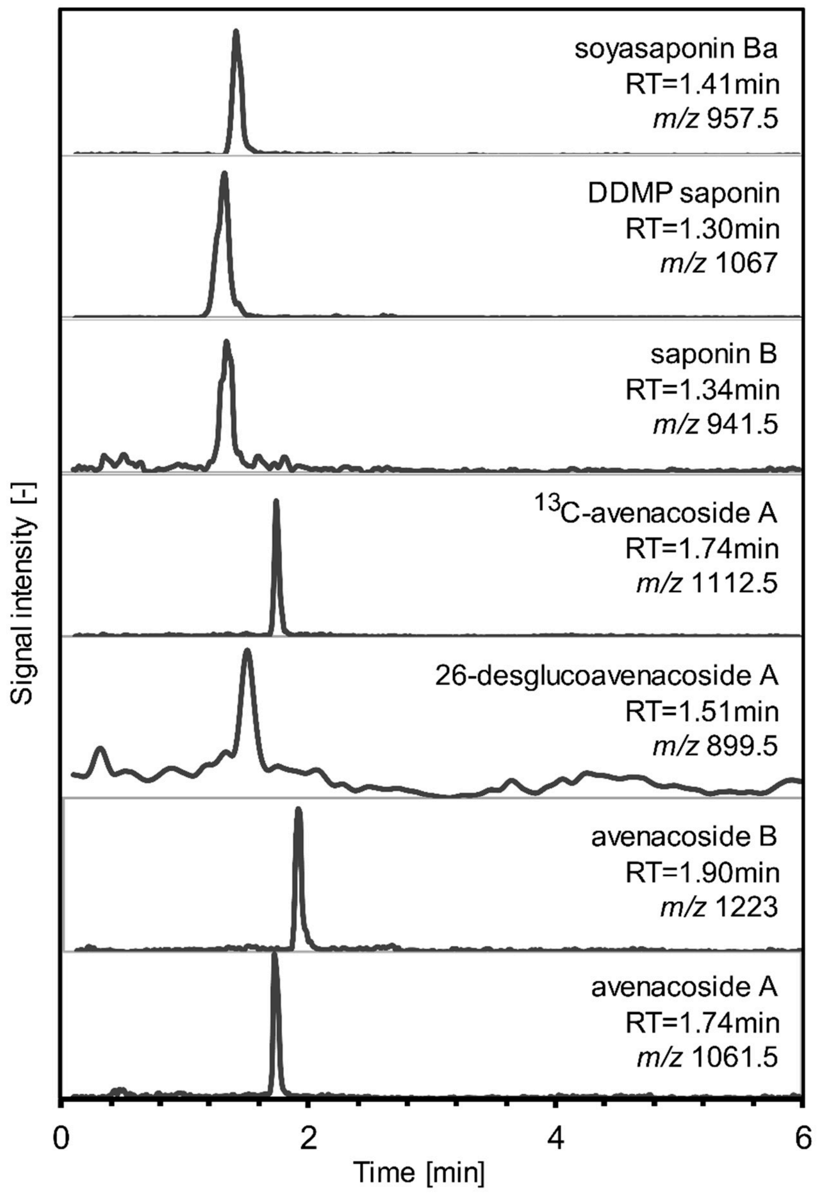

Figure S1 LC-MS chromatograms of oat and pea flours (SIR and ESI-). In oat flour: avenacoside A, avenacoside B, 26-desglucoavenacoside A and internal standard ^{13}C-avenacoside A. In pea flour: saponin B, DDMP saponin and internal standard soyasaponin Ba

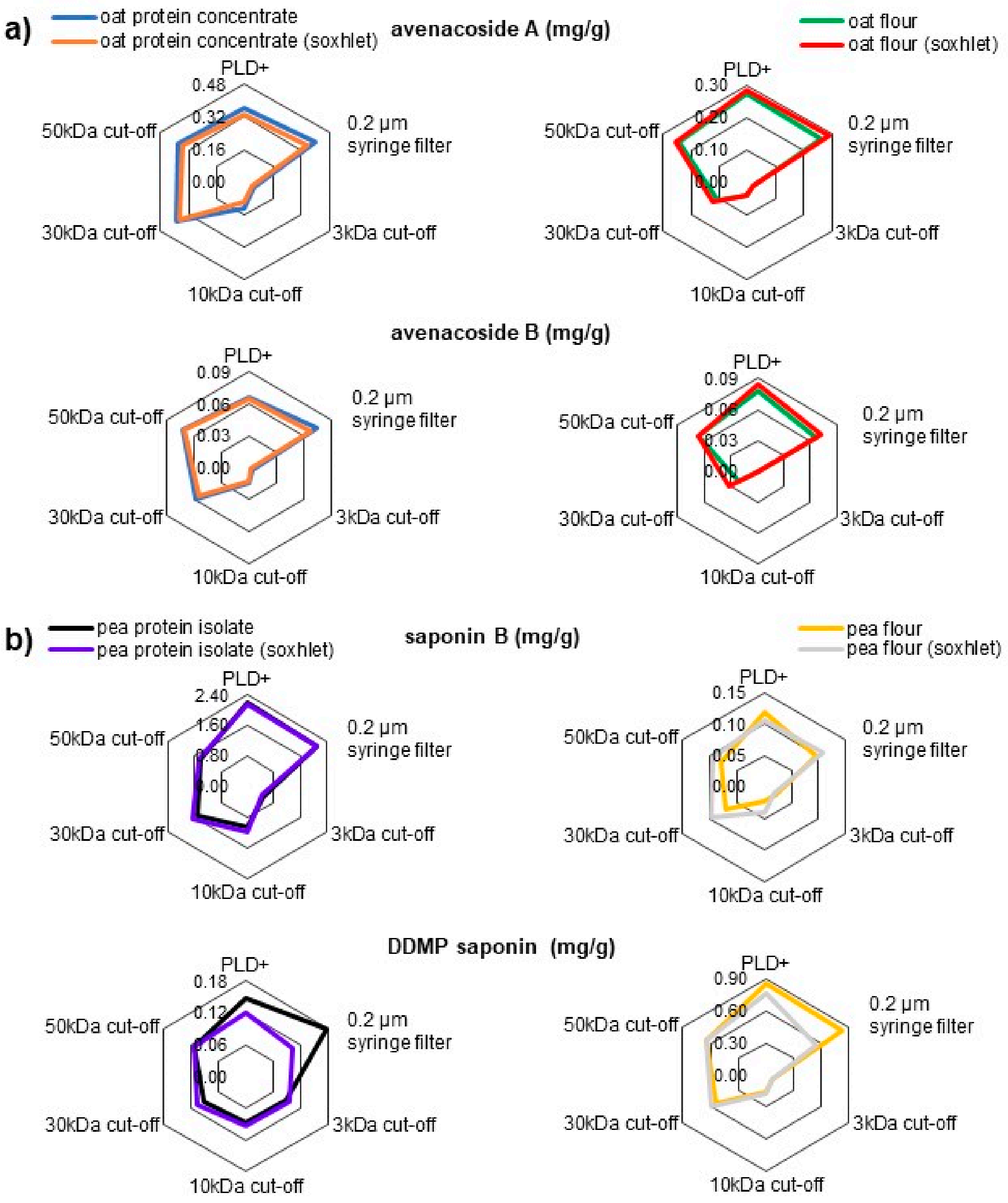

Figure S2 Saponin yield in (a) oat and (b) pea matrices – the effect of sample clean-up: the pre-extraction of fat and six post-extraction filtration possibilities. The results of avenacoside B are presented in equivalents of avenacoside A mg/g and DDMP saponin in equivalents of saponin B mg/g. Each result represents mean of two replicates.

Table S2 The effectiveness of ultrasonic bath extraction compared to reference extraction conditions using the tube rotator (extraction yield 100%)[a]

		**yield (%)**	
**matrix**	analyte	**flour**	**protein concentrate/isolate**
**oat**	avenacoside A	98 ± 2	101 ± 0.5
	avenacoside B	96 ± 6	100 ± 2
**pea**	saponin B	104 ± 3	109 ± 6
	DDMP saponin	108 ± 6	110 ± 21[b]

[a] Each result represents mean ± standard deviation (n = 2)

[b] value is close to LOQ

REFERENCES

Heng L, Vincken J-P, Koningsveld G van, et al (2006) Bitterness of saponins and their content in dry peas. J Sci Food Agric 86:1225–1231. https://doi.org/10.1002/jsfa.2473

Pecio Ł, Wawrzyniak-Szołkowska A, Oleszek W, Stochmal A (2013) Rapid analysis of avenacosides in grain and husks of oats by UPLC–TQ–MS. Food Chem 141:2300–2304. https://doi.org/10.1016/j.foodchem.2013.04.094
